# Effect of hydroalcoholic *Echium amoenum* extract on scopolamine-induced learning and memory impairment in rats

**DOI:** 10.1080/13880209.2018.1543330

**Published:** 2018-12-12

**Authors:** Zahra Rabiei, Mahbubeh Setorki

**Affiliations:** a Medical Plants Research Center, Basic Health Sciences Institute, Shahrekord University of Medical Sciences, Shahrekord, Iran;; b Department of Biology, Izeh Branch, Islamic Azad University, Izeh, Iran

**Keywords:** Cognitive performance, locomotor activity, anxiety

## Abstract

**Context:** Scopolamine, a muscarinic receptor antagonist, causes memory loss that resembles Alzheimer's disease (AD). *Echium amoenum* L. (Boraginaceae) is a famous medicinal plant of Iran that is traditionally used as a sedative and mood enhancer.

**Objective:** This study evaluates the effect of hydroalcoholic extract of *E. amoenum* flowers on scopolamine-induced memory impairment in rats.

**Materials and methods:** Fifty male Wistar rats were randomly divided into five groups. Control group received normal saline, model group received scopolamine (0.7 mg/kg, IP, daily for 21 days), and test groups received *E. amoenum* extract (50, 75, and 100 mg/kg, IP, daily for 21 days) 30 min before each scopolamine injection. The elevated plus maze (EPM), shuttle box, novel object and rotarod tests were performed after treatment. Brain levels of malondialdehyde (MDA) and total antioxidant capacity (TCA) were also determined.

**Results:** Scopolamine-treated rats spent more time exploring the novel object compared to the control, and *E. amoenum* extract at all three doses significantly decreased the time spent exploring the novel object (*p* < 0.05). *E. amoenum* extract (75 and 100 mg/kg) significantly elongated the secondary latency in rats receiving scopolamine in the shuttle box test (*p* < 0.05). In addition, treatment with 75 and 100 mg/kg doses of *E. amoenum* extract significantly ameliorated scopolamine-induced motor in coordination in rotarod test (*p* < 0.05). It also significantly increased the time spent in the open arms and reduced the time spent in the closed arms of EPM (*p* < 0.05). Treatment of scopolamine-exposed rats with *E. amoenum* extract significantly increased TCA and reduced MDA level of brain (*p* < 0.05).

**Discussion and conclusions:***E. amoenum* extract shows protective effect against scopolamine-induced impairment and is suggested to be tested in clinical trials to evaluate the efficacy on AD.

## Introduction

The prevalence of neurodegenerative diseases, such as Alzheimer's disease (AD) and Parkinson's disease associated with cognitive and motor impairment, is one of the consequences of the ageing phenomenon of the world's population (Kumar and Singh 2015). AD is a cerebrovascular disorder that gradually decreases the mental abilities of the patient (Kumar and Singh 2015). Although the main causes of the disease are uncertain, several factors such as accumulation of β-amyloid and tau proteins, oxidative stress, brain inflammation, as well as inhibition of cholinergic receptors and decreased cholinergic activity can be a cause for cognitive impairment in AD (Dolatabadi et al. 2010).

Scopolamine is a muscarinic receptor antagonist, which causes temporary memory impairment and a pattern similar to that of AD in animals (Caine et al. 1981). Research has shown that consuming different drugs can improve the cognitive and functional capacity of AD patients, but may also lead to unexpected side effects (Braak and Braak 1991; Grover et al. 2012). Regarding the high prevalence of the central nervous system diseases, including AD, and lack of definitive treatment for these diseases, as well as the side effects and inadequate efficacy of chemical drugs, there are studies seeking to find ingredients with satisfactory efficacy (Ahmad et al. 2014).

*Echium amoenum* L. (Boraginaceae) is an annual herbaceous plant that occurs spontaneously and exclusively in the hillsides of Alborz Mountain Range in Iran and cannot be cultivated. The plant reaches a height of, at most, 60–100 cm, and its flowers are blue, pink, red or purple, which, after drying, change into dark purple or blue (Heidari et al. 2006). *E. amoenum* is one of the most important medicinal plants in Iranian traditional medicine, and its tea is one of the most commonly used herbal drugs. All parts of the plant, except for root, including stems, leaves and flowers have pharmaceutical uses (Asadi et al. 2004). *E. amoenum* is widely used as a sedative and mood enhancer. It is also used to treat cough, sore throat and pneumonia (Behnammanesh et al. 2015).

Neuroprotective effects of *E. amoenum,* including anti-ischemic (Safaeian et al. 2015), analgesic (Heidari et al. 2006) and anxiolytic effects (Rabbani et al. 2004), have been shown in animal models. Recent studies have suggested that the aqueous extract of *E. amoenum* is effective in treating patients with mild to moderate depression (Asadi et al. 2004), as well as obsessive-compulsive disorder (Asadi et al. 2004), and generalized anxiety disorder (Sayyah et al. 2012). Bluish-purple flowers of *E. amoenum* are known as one of the most important sources of phenolic compounds such as rosmarinic acid, cyanidin and delphinidin (Safaeian et al. 2015). Cyanidin-3-glucoside, the most important anthocyanin in the plant, exhibits protective effects against brain damage and apoptosis caused by cerebral ischemia (Min et al. 2011). According to the articles mentioned above, it seems that *E. amoenum* is effective against cognitive impairments caused by neurodegenerative diseases. Therefore, this study evaluates the effect of *E. amoenum* extract on scopolamine-induced memory impairment in rats.

## Materials and methods

### Preparation of E. amoenum extract

The flowers of *E. amoenum,* were purchased from a local market of Shahrekord, Iran in July 2017 and identified by Dr Rafieian, herbalist, and then a reference sample was kept in the Herbarium of Islamic Azad University of Izeh (voucher No. 549). The extraction was conducted by a maceration method. The dried plant sample (1 kg) was pulverized by an electric mill and then mixed with 70% ethanol at 1:5 sample/solvent ratio (w/v). After 72 h, the solution was filtered through Whatman filter paper No. 1 and the filtrate evaporated under vacuum at 40 °C to dryness. Finally, the resulting solution was completely dried at 37 ± 1 °C. The percentage of crude hydroalcoholic extract yield from the dried flowers of *E. amoenum* was 3% (w/w).

### Grouping and treatment of animals

Male Wistar rats weighing 200–250 g were kept in controlled conditions (21 ± 2 °C, 12 h light/dark cycle) with free access to water and food. Rats were then randomly assigned into five groups of 10 each. The control group received normal saline (1 mL/kg) for 21 days. The scopolamine group intraperitoneally received scopolamine at a dose of 0.7 mg/kg for 21 days. Extract treated groups intraperitoneally received scopolamine and then *E. amoenum* extract at 50, 75, and 100 mg/kg for 21 days. Behavioural tests were conducted for several days from day 21. The selection of the doses of scopolamine and *E. amoenum* extract based on previously published studies (Asgharzade et al. 2015; Safaeian et al. 2015). After behavioural tests, rat underwent general anesthesia using Chloral hydrate (800 mg/kg) and sample of brain tissue was obtained and stored at -70 °C for biochemical assays.

### Shuttle box test

Passive avoidance memory was measured by shuttle box. This apparatus has a bright chamber connected to a dark chamber by a guillotine door. Electric shocks are exerted to a conductive metal grid on the floor of the apparatus by a separate stimulus. This test was performed on each rat for four consecutive days. On the first two days, rats were individually allowed to freely explore the apparatus for 5 min. On the third day, an acquisition test was conducted. Rats were left in the bright chamber and, after 2 min acclimatization, the guillotine door was opened and after the animal entry into the dark chamber, it was closed and an electrical shock (1 mA/s) was exerted to it and the latency to enter the dark chamber was recorded as initial latency. Twenty-four hours later, each rat was placed in the bright chamber and latency to enter the dark chamber was measured as secondary latency (up to 60 s) (Rabiei et al. 2015).

#### Novel object test

In novel object test, animals were tested using a 50 × 25 × 50 × 25 cm open black box for two consecutive days. On the first day, animals were placed in the apparatus without objects and permitted to explore for 5 min. On the second day, two equal objects were presented for exploration (T_1_). Object exploration was defined as sniffing or touching the object at <2 cm from the nose or exploring both the objects for ≥10 s. After identification, T_1_ trial was terminated and animal was taken back to its home cage. T_2_ trial was performed 24 h later. In this step, animal was permitted to freely explore a novel and familiar object for 4 min. In order to reduce the effect of place and object preference, two objects were randomly placed in the apparatus. The time spent to explore the new and familiar objects was rerecorded on video trap (Karasawa et al. 2008).

### Rotarod performance test

The ability to maintain balance and motor resistance was investigated using rotarod. This apparatus has a rod that rotates at a speed of 0–40 rpm. The apparatus also has a belt and the speed of the rod can be adjusted by changing the belt position. First, the animal was placed on the rotating rod of the apparatus and trained to walk on it according to the main protocol (10 rpm and 7 rpm^2^ acceleration). Thirty minutes later, the rat was again placed on the rod and the time to maintain balance and resist rod movement was recorded. The maximum time for each animal in this test was considered to be 300 s (Asgharzade et al. 2015).

### Elevated plus maze (EPM) test

An apparatus called elevated plus maze was used to measure anxiety. This apparatus has two opposite open arms, two opposite closed arms, and a central sheath elevated 50 cm above the floor. This test was performed in a relatively dark, silent chamber, and each animal was placed gently in the center of the device facing the open arm and allowed to explore for 5 min. The number of entries and time spent in each arm were recorded (Lee et al. 2014).

### Measuring total antioxidant capacity (TCA)

Total antioxidant capacity of brain tissue homogenate was determined by the Ferric ion reducing antioxidant power (FRAP). The FRAP solution was prepared by adding 2.5 mL of 0.25 mM acetate buffer with pH 3, 2.5 mL of 10 mM 2,4,6-Tris(2-pyridyl)-s-triazine (TPTZ), prepared in 40 mM hydrochloric acid, and 2.5 mL of 20 mM 6 H_2_O FeCl. The tissue homogenate sample (25 μL) was mixed with 1.5 mL of FRAP working solution, and the optical absorbance of the resulting mixture was read by the spectrophotometer at 593 nm, after it was left at 37 °C for 10 min (El-Sherbiny et al. 2003).

### Measuring malondialdehyde (MDA) level

Brain tissue homogenate (25 μL) was mixed with 1.5 mL of acetic acid 20%, 1.5 mL of 0.8 mM thiobarbituric acid (TBA), and 200 μL of SDS 1.8% solution. The sample was then placed in boiling water for 60 min. The sample was cooled and 1 mL of distilled water and 5 mL of *n*-butanol-pyridine solution added to it and the resulting mixture was shaken. The mixture was then centrifuged for 10 min at 4000 rpm and the optical absorbance of the supernatant was recorded at 523 nm (Ben-Nasr et al. 2015).

### Data analysis

Data were entered into the SPSS version 21 (SPSS lnc. Chicago, IL, USA). One-way ANOVA followed by Tukey's test was used to analyse the data. The data were expressed as mean ± standard deviation (SD) and *p* < 0.05 was considered significance level.

## Results

**Figure 1.**
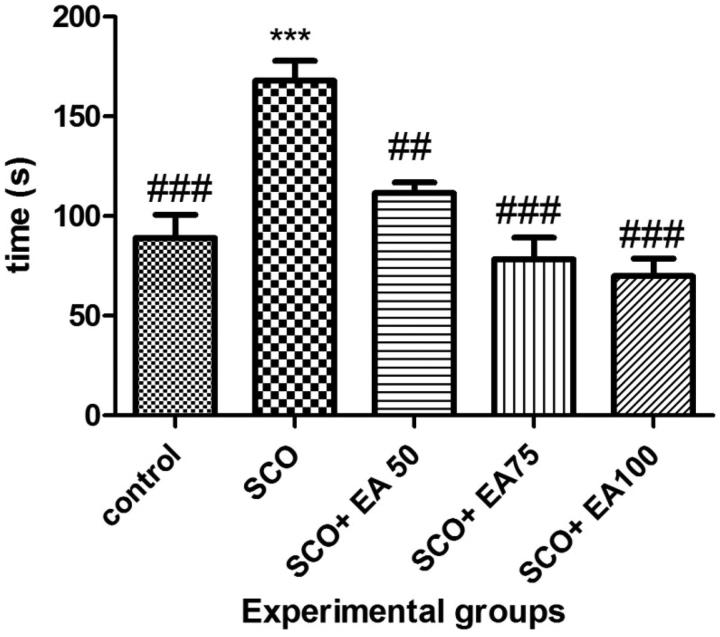
Comparison of the results of novel object recognition test between groups. * Shows significant differences with control group (***p < 0.001). ^#^ Shows significant differences with scopolamine treated group (^###^*p* < 0.001, ^##^*p* < 0.01). SCO = Scopolamine, SCO + EA 50, 75 and 100 = scopolamine plus *E. amoenum* extract at doses of 50, 75 and 100 mg/kg.

The results from the novel object identification test are illustrated in Figure 1. According to the results, the time taken to identify the new object was significantly higher in the scopolamine group than in the controls (*p* < 0.001). Treatment with *E. amoenum* extract at 50, 75 and 100 mg/kg in rats given scopolamine caused a significant decrease in the time taken to identify the new object when compared to the scopolamine receiving rats (*p* < 0.001 and *p* < 0.01, respectively).

**Figure 2.**
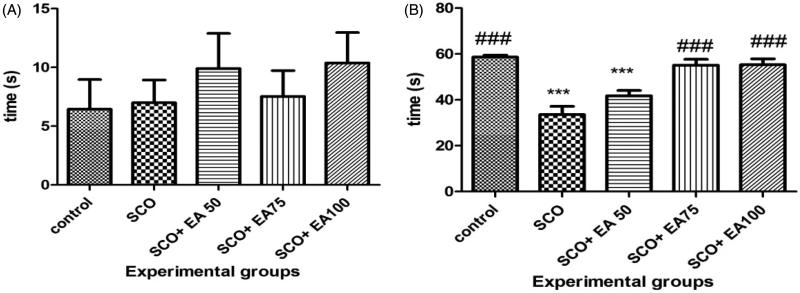
Comparison of the initial (a) and secondary (b) latency time in shuttle box test between groups. *Shows significant differences with control group (***p < 0.001). ^#^ Shows significant differences with scopolamine treated group (^###^*p* < 0.001). SCO = Scopolamine, SCO + EA 50, 75 and 100 = scopolamine plus *E. amoenum* extract at doses of 50, 75 and 100 mg/kg.

The results of the initial and secondary latencies in the passive avoidance task are illustrated in Figure 2. According to the results, there was no significant difference in the initial latency to enter the dark chamber between the groups. Secondary latency time was significantly lower in the scopolamine receiving group than in the control group (*p* < 0.001). Treatment of rats receiving scopolamine with *E. amoenum* extract at 75 and 100 mg/kg caused a significant increase in secondary latency time (*p* < 0.001).

**Figure 3.**
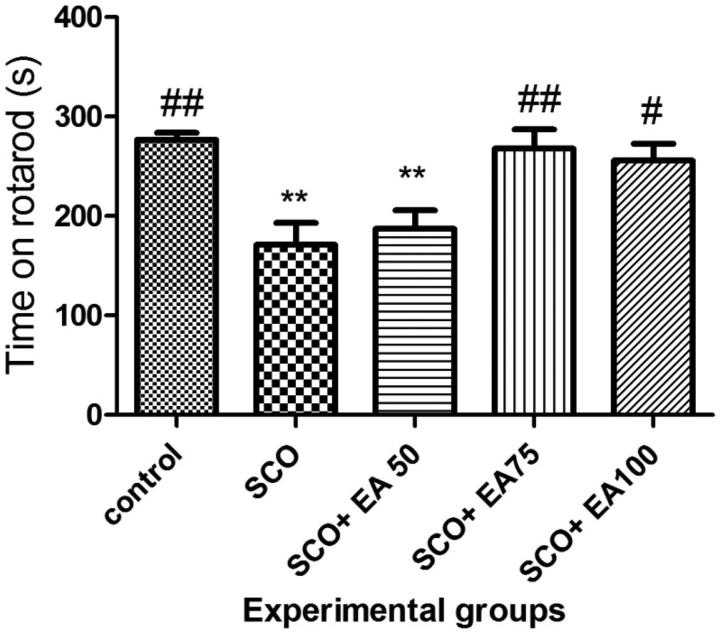
Comparison of the balance maintenance time in rotarod test between groups. * Shows significant differences with control group (**p < 0.01). ^#^ Shows significant differences with scopolamine treated group (^##^*p* < 0.001, ^#^*p* < 0.05). SCO = Scopolamine, SCO + EA 50, 75 and 100 = scopolamine plus *E. amoenum* extract at doses of 50, 75 and 100 mg/kg.

The results on the duration of the balance in the rotarod test in different groups are illustrated in Figure 3. The duration of balance in the scopolamine group was significantly lower than that in the control group (*p* < 0.01) and the treatment with the extract of *E. amoenum* at 75 and 100 mg/kg caused a significant improvement of the balance duration (*p* < 0.01 and *p* < 0.05, respectively).

**Figure 4.**
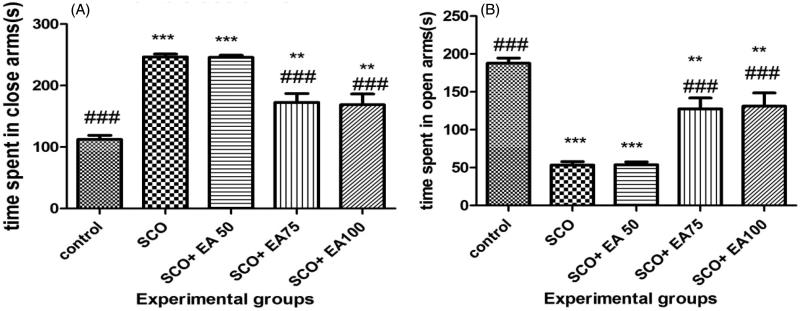
Comparison of the time spent in the closed (a) and open (b) arms of the elevated plus maze (EPM) between groups. * Shows significant differences with control group (***p < 0.001, **p < 0.01). ^#^ Shows significant differences with scopolamine treated group (^###^*p* < 0.001). SCO = Scopolamine, SCO + EA 50, 75 and 100 = scopolamine plus *E. amoenum* extract at doses of 50, 75 and 100 mg/kg.

As illustrated in Figure 4, scopolamine exposure significantly increased time spent in the closed arms and reduced time spent in the open arms of elevated plus maze (*p* < 0.001). Treatment of rats given scopolamine with *E. amoenum* extract at 75 and 100 mg/kg significantly reduced the time spent in the closed arms and increased the time spent in the open arms (*p* < 0.001).

As the results in Table 1 show, in rats treated with scopolamine a significant increase in the brain MDA level was noted (*p* < 0.001). Administration of *E. amoenum* extract at 75 and 100 mg/kg significantly reduced the brain MDA as compared with the scopolamine-treated group (*p* < 0.001).

**Table 1.** Comparison of brain malondialdehyde (MDA) levels between groups.

Groups	MDA(µmol/g)
Control	132.85 ± 32.05
SCO	342.25 ± 81.64***
SCO + EA 50	287.00 ± 52.24
SCO + EA 75	162.42 ± 15.30^###^
SCO + EA 100	144.00 ± 44.08^###^

* Shows significant differences with control group (***p < 0.001). ^#^ Shows significant differences with scopolamine treated group (^###^*p* < 0.001).

SCO = Scopolamine, SCO + EA 50, 75 and 100 = scopolamine plus *E. amoenum* extract at doses of 50, 75 and 100 mg/kg.

As the results in Table 2 show, the treatment of rats with scopolamine significantly reduced the antioxidant capacity of brain (*p* < 0.001). The antioxidant capacity of brain showed significant improvement in animal treated with *E. amoenum* extract at 50, 75 and 100 mg/kg (*p* < 0.001).

**Table 2.** Comparison of brain total antioxidant capacity (TCA) between groups.

Groups	TCA (µmol/g)
Control	859.64 ± 70.05
SCO	215.99 ± 44.08***
SCO + EA 50	550.97 ± 64.81^###^
SCO + EA 75	556.45 ± 24.52^###^
SCO + EA 100	711.55 ± 30.15^###^

* Shows significant differences with control group (***p < 0.001). ^#^ Shows significant differences with scopolamine treated group (^###^*p* < 0.001).

SCO = Scopolamine, SCO + EA 50, 75 and 100 = scopolamine plus *E. amoenum* extract at doses of 50, 75 and 100 mg/kg.

## Discussion

In the present study, the protective effects of hydroalcoholic extract of *E. amoenum* on scopolamine-induced learning and memory impairments were investigated. Treatment of rats by sequential injections of scopolamine for 21 days resulted in impaired memory function accompanied by shorter step through latency in the shuttle box and lower new object exploration time in the novel object test. It was previously shown that scopolamine exposure impairs memory and learning capacity in shuttle box and also novel object test (Bartolini et al. 1996; Seifhosseini et al. 2011). Scopolamine, a muscarinic receptor antagonist, readily passes the blood brain barrier to induce anti-muscarinic activity leading to cholinergic deficit and memory loss. As an age-related decline in memory function is thought to be prominently due to impairment of cholinergic neurotransmission, scopolamine exposure has often been applied to induce experimental dementia to assess new drugs for learning and memory impairments (Stone et al. 1988, 1991). Our data demonstrated that extract of *E. amoenum* shows potent memory enhancing effects in both shuttle box and novel object tests. To our knowledge, there was no previous study on memory-enhancing activity of *E. amoenum*, but rosmarinic acid which found in this plant shows considerable cholinergic activity and improves scopolamine-induced memory loss (Hasanein and Mahtaj 2015).

In our study, scopolamine exposure also induced anxiety like behaviours in the elevated plus maze and reduced balance in the rotarod test which is in judgement with previous studies (Rahmati et al. 2017). In our study, *E. amoenum* extract at 75 and 100 mg/kg significantly ameliorated anxiety-like behaviours and improved ability to maintain balance on the rotarod. Consistent with the present results, the anti-anxiety effects of *E. amoenum* were shown in the mouse model (Shafaghi et al. 2010) and also double-blind clinical trial (Shafaghi et al. 2010; Sayyah et al. 2012).

In the present study, scopolamine injection caused a significant reduction in the antioxidant capacity of brain and a significant increase in its MDA, as the index of lipid peroxidation. It is well known that scopolamine causes memory impairment by attenuating cholinergic neurotransmission, as well as increases of markers of oxidative stress in the brain (Budzynska et al. 2015). Experimental studies have found that injection of scopolamine in rats induces the peroxidation of lipids and weakens intrinsic antioxidant defenses in whole brain, as well as in regions associated with memory and learning process (Kwon et al. 2010). Brain is particularly vulnerable to oxidative stress due to the high content of highly oxidizable polyunsaturated fatty acids and the relatively low antioxidant defenses in comparison with other tissues. During oxidative stress, overproduced ROS can react with vital cellular components such as proteins, nucleic acids, lipids and carbohydrates leading to neuronal cell damage and apoptosis, and ultimately impaired memory and learning function (Markesbery 1997). In our study, *E. amoenum* extract treatment significantly attenuated thescopolamine-induced lipid peroxidation and reduced antioxidant capacity. Antioxidant function of *E. amoenum* has been previously reported. In a study of 37 healthy volunteers, daily consumption of *E. amoenum* at 7 mg/kg enhanced serum antioxidant capacity and significantly decreased lipid peroxidation (Ranjbar et al. 2006). The antioxidant effects of *E. amoenum* have been attributed to the presence of phenolic compounds flavonoids, β-carotene, vitamin C, anthocyanins, and tannins (Pilerood and Prakash 2014). It has been reported that one of the main phenolic compounds of the plant, rosmarinic acid, exhibits protective effects against oxidative damage of dopaminergic neurons caused by hydrogen peroxide (Lee et al. 2008). Cyanidin and delphinidin, found in abundance in *E. amoenum* extract, decrease the production of hydrogen peroxide and increase the activity of glutathione reductase and glutathione content in cell culture media (Cvorovic et al. 2010). Accordingly, it can be argued that *E. amoenum* extract can prevent oxidative damage to the neurons and subsequent memory function degradation.

Inflammatory processes play an important role in the pathogenesis of degenerative changes and cognitive impairment associated with AD (Heneka et al. 2015). Brain inflammatory markers [cyclooxygenase 1 (COX-1), cyclooxygenase 2 (COX-2), IL-1β, and IL-10)] have been reported to increase in scopolamine-induced memory loss (Ahmad et al. 2014). Scopolamine activates the NF-κB pathway in neurons. This transcription factor can increase the expression of genes such as TNF-α, iNOS and COX-2 (Jang et al. 2013). In addition, studies have shown that anti-inflammatory drugs have a positive effect on cognitive function in mice treated with lipopolysaccharide and scopolamine (Jain et al. 2002) Cyanidin-3 glucoside, one of the most abundant anthocyanin in *E. amoenum* extract reduces the production of inflammatory agents such as prostaglandin E_2_, and COX-2 in cancer cells by inhibiting the activation of the transcription factor C-jun and NF-κB (Munoz-Espada and Watkins 2006). Delphinidin, another anthocyanin of the plant, directly inhibits the activity of kinase Fyn and thus reduces TNF-α-induced COX-2 expression (Hwang et al. 2009). Therefore, due to the anti-inflammatory activity of *E. amoenum* and its active ingredients, inflammatory mechanisms can be considered to be the major mechanisms involved in the plant activity, although it is necessary to further investigate this argument in future studies.

*Echium amoenum* is used in Iranian traditional medicine to reduce anxiety and tension and produce calming effects. However, few studies have been conducted on neuroprotective effects of the plant in animal models as well as clinical trials. There is very limited information on plant active ingredients and the mechanism of neuroprotective effects, which requires further research in this regard.

## Conclusions

The present study indicated the efficacy of *E. amoenum* extract in improving scopolamine-induced memory impairment, anxiety and imbalance via antioxidant activity and reducing brain lipid peroxidation. Therefore, an investigation on cognitive functions, motor imbalance, and psychiatric disorders in AD patients is recommended.

## References

[CIT0001] Ahmad A, Ramasamy K, Jaafar SM, Majeed ABA, Mani V 2014 Total isoflavones from soybean and tempeh reversed scopolamine-induced amnesia, improved cholinergic activities and reduced neuroinflammation in brain. Food Chem Toxicol. 65:120–128. 24373829 10.1016/j.fct.2013.12.025

[CIT0002] Asadi S, Amini H, Akhoundzadeh S, Saiiah M, Kamalinezhad M 2004 Efficacy of aqueous extract of *Echium amoenum* L. in the treatment of mild to moderate major depressive disorder: a randomized double blind clinical trial. J Med Plants. 3:61–70.

[CIT0003] Asgharzade S, Rabiei Z, Rafieian-Kopaei M 2015 Effects of *Matricaria chamomilla* extract on motor coordination impairment induced by scopolamine in rats. Asian Pac J Trop Biomed. 5:829–833.

[CIT0004] Bartolini L, Casamenti F, Pepeu G 1996 Aniracetam restores object recognition impaired by age, scopolamine, and nucleus basalis lesions. Pharmacol Biochem Behav. 53:277–283. 8808132 10.1016/0091-3057(95)02021-7

[CIT0005] Behnammanesh G, Khalilpour S, Majid ASA, Majid AMSA 2015 Pharmacological actions and potential neuroprotective effects of *Rhus coriaria* L. And *Echium amoenum* L.: a brief review. Pharmacology. 11:14–21.

[CIT0006] Ben-Nasr S, Aazza S, Mnif W, Miguel MGC 2015 Antioxidant and anti-lipoxygenase activities of extracts from different parts of *Lavatera cretica* L. grown in Algarve (Portugal). Pharmacognosy Mag. 11:48. 10.4103/0973-1296.149743 PMC4329632 25709210

[CIT0007] Braak H, Braak E 1991 Neuropathological stageing of Alzheimer-related changes. Acta Neuropathol. 82:239–259. 1759558 10.1007/BF00308809

[CIT0008] Budzynska B, Boguszewska-Czubara A, Kruk-Slomka M, Skalicka-Wozniak K, Michalak A, Musik I, Biala G 2015 Effects of imperatorin on scopolamine-induced cognitive impairment and oxidative stress in mice. Psychopharmacol. 232:931–942. 10.1007/s00213-014-3728-6 PMC4325182 25189792

[CIT0009] Caine ED, Weingartner H, Ludlow CL, Cudahy EA, Wehry S 1981 Qualitative analysis of scopolamine-induced amnesia. Psychopharmacol. 74:74–80. 10.1007/BF00431761 6791209

[CIT0010] Cvorovic J, Tramer F, Granzotto M, Candussio L, Decorti G, Passamonti S 2010 Oxidative stress-based cytotoxicity of delphinidin and cyanidin in colon cancer cells. Arch Biochem Biophys. 501:151–157. 20494645 10.1016/j.abb.2010.05.019

[CIT0011] Dolatabadi HRD, Reisi P, Malekabadi HRA, Alaei H, Pilehvarian AA 2010 Effects of folic acid on passive avoidance learning and memory in rat Alzheimer model by intracerebroventricular injection of streptozotocin. J Isfahan Med Sch. 28:776–783.

[CIT0012] El-Sherbiny DA, Khalifa AE, Attia AS, Eldenshary EE-DS 2003 *Hypericum perforatum* extract demonstrates antioxidant properties against elevated rat brain oxidative status induced by amnestic dose of scopolamine. Pharmacol Biochem Behav. 76:525–533. 14643852 10.1016/j.pbb.2003.09.014

[CIT0013] Grover A, Shandilya A, Agrawal V, Bisaria VS, Sundar D 2012 Computational evidence to inhibition of human acetylcholinesterase by withanolide A for Alzheimer treatment. J Biomolec Struct Dynam. 29:651–662. 10.1080/07391102.2012.10507408 22208270

[CIT0014] Hasanein P, Mahtaj AK 2015 Ameliorative effect of rosmarinic acid on scopolamine-induced memory impairment in rats. Neurosci Lett. 585:23–27. 25445372 10.1016/j.neulet.2014.11.027

[CIT0015] Heidari MR, Azad EM, Mehrabani M 2006 Evaluation of the analgesic effect of *Echium amoenum* Fisch & CA Mey. extract in mice: possible mechanism involved. J Ethnopharmacol. 103:345–349. 16185831 10.1016/j.jep.2005.08.027

[CIT0016] Heneka MT, Carson MJ, El Khoury J, Landreth GE, Brosseron F, Feinstein DL, Jacobs AH, Wyss-Coray T, Vitorica J, Ransohoff RM, et al. 2015 Neuroinflammation in Alzheimer's disease. Lancet Neurol. 14:388–405. 25792098 10.1016/S1474-4422(15)70016-5 PMC5909703

[CIT0017] Hwang MK, Kang NJ, Heo Y-S, Lee KW, Lee HJ 2009 Fyn kinase is a direct molecular target of delphinidin for the inhibition of cyclooxygenase-2 expression induced by tumor necrosis factor-alpha. Biochem Pharmacol. 77:1213–1222. 19174152 10.1016/j.bcp.2008.12.021

[CIT0018] Jain NK, Patil C, Kulkarni SK, Singh A 2002 Modulatory role of cyclooxygenase inhibitors in aging- and scopolamine or lipopolysaccharide-induced cognitive dysfunction in mice. Behav Brain Res. 133:369–376. 12110471 10.1016/s0166-4328(02)00025-6

[CIT0019] Jang YJ, Kim J, Shim J, Kim C-Y, Jang J-H, Lee KW, Lee HJ 2013 Decaffeinated coffee prevents scopolamine-induced memory impairment in rats. Behav Brain Res. 245:113–119. 23415910 10.1016/j.bbr.2013.02.003

[CIT0020] Karasawa J-I, Hashimoto K, Chaki S 2008 D-Serine and a glycine transporter inhibitor improve MK-801-induced cognitive deficits in a novel object recognition test in rats. Behav Brain Res. 186:78–83. 17854919 10.1016/j.bbr.2007.07.033

[CIT0021] Kumar A, Singh A 2015 A review on Alzheimer's disease pathophysiology and its management: an update. Pharmacol Rep. 67:195–203. 25712639 10.1016/j.pharep.2014.09.004

[CIT0022] Kwon S-H, Lee H-K, Kim J-A, Hong S-I, Kim H-C, Jo T-H, Park Y-I, Lee C-K, Kim Y-B, Lee S-Y, et al. 2010 Neuroprotective effects of chlorogenic acid on scopolamine-induced amnesia via anti-acetylcholinesterase and anti-oxidative activities in mice. Eur J Pharmacol. 649:210–217. 20854806 10.1016/j.ejphar.2010.09.001

[CIT0023] Lee B, Sur B, Shim J, Hahm D-H, Lee H 2014 Acupuncture stimulation improves scopolamine-induced cognitive impairment via activation of cholinergic system and regulation of BDNF and CREB expressions in rats. BMC Complement Altern Med. 14:338. 25231482 10.1186/1472-6882-14-338 PMC4180318

[CIT0024] Lee HJ, Cho H-S, Park E, Kim S, Lee S-Y, Kim C-S, Kim DK, Kim S-J, Chun HS 2008 Rosmarinic acid protects human dopaminergic neuronal cells against hydrogen peroxide-induced apoptosis. Toxicol. 250:109–115. 10.1016/j.tox.2008.06.010 18644421

[CIT0025] Markesbery WR 1997 Oxidative stress hypothesis in Alzheimer's disease. Free Radical Biol Med. 23:134–147. 9165306 10.1016/s0891-5849(96)00629-6

[CIT0026] Min J, Yu S-W, Baek S-H, Nair KM, Bae O-N, Bhatt A, Kassab M, Nair MG, Majid A 2011 Neuroprotective effect of cyanidin-3-*O*-glucoside anthocyanin in mice with focal cerebral ischemia. Neurosci Lett. 500:157–161. 21651957 10.1016/j.neulet.2011.05.048

[CIT0027] Munoz-Espada AC, Watkins BA 2006 Cyanidin attenuates PGE2 production and cyclooxygenase-2 expression in LNCaP human prostate cancer cells. J Nutr Biochem. 17:589–596. 16443360 10.1016/j.jnutbio.2005.10.007

[CIT0028] Pilerood S, Prakash J 2014 Evaluation of nutritional composition and antioxidant activity of borage (*Echium amoenum*) and valerian (*Valerian officinalis*). J Food Sci Technol. 51:845–856. 24803690 10.1007/s13197-011-0573-z PMC4008743

[CIT0029] Rabbani M, Sajjadi S, Vaseghi G, Jafarian A 2004 Anxiolytic effects of *Echium amoenum* on the elevated plus-maze model of anxiety in mice. Fitoterapia. 75:457–464. 15261383 10.1016/j.fitote.2004.04.004

[CIT0030] Rabiei Z, Mokhtari S, Asgharzade S, Gholami M, Rahnama S, Rafieian-Kopaei M 2015 Inhibitory effect of *Thymus vulgaris* extract on memory impairment induced by scopolamine in rat. Asian Pac J Trop Biomed. 5:845–851.

[CIT0031] Rahmati B, Kiasalari Z, Roghani M, Khalili M, Ansari F 2017 Antidepressant and anxiolytic activity of *Lavandula officinalis* aerial parts hydroalcoholic extract in scopolamine-treated rats. Pharmaceutl Biol. 55:958–965. 10.1080/13880209.2017.1285320 PMC6130744 28166686

[CIT0032] Ranjbar A, Khorami S, Safarabadi M, Shahmoradi A, Malekirad AA, Vakilian K, Mandegary A, Abdollahi M 2006 Antioxidant activity of Iranian *Echium amoenum* Fisch & CA Mey flower decoction in humans: a cross-sectional before/after clinical trial. Evid Based Complement Alternat Med. 3:469–473. 17173110 10.1093/ecam/nel031 PMC1697746

[CIT0033] Safaeian L, Tameh AA, Ghannadi A, Naghani EA, Tavazoei H, Alavi SS 2015 Protective effects of *Echium amoenum* Fisch. and CA Mey. against cerebral ischemia in the rats. Adv Biomed Res. 4:22–29. 26261809 10.4103/2277-9175.157809 PMC4513330

[CIT0034] Sayyah M, Siahpoosh A, Khalili H, Malayeri A, Samaee H 2012 A double-blind, placebo-controlled study of the aqueous extract of *Echium amoenum* for patients with general anxiety disorder. Iran J Pharm Res. 11:697. 24250495 PMC3832167

[CIT0035] Seifhosseini S, Jahanshahi M, Moghimi A, Aazami N-S 2011 The effect of scopolamine on avoidance memory and hippocampal neurons in male Wistar rats. Basic Clin Neurosci. 3:9–15.

[CIT0036] Shafaghi B, Naderi N, Tahmasb L, Kamalinejad M 2010 Anxiolytic effect of *Echium amoenum* L. in mice. Iran J Pharm Res. 145:37–41.

[CIT0037] Stone WS, Croul CE, Gold PE 1988 Attenuation of scopolamine-induced amnesia in mice. Psychopharmacol. 96:417–420. 10.1007/BF00216073 3146778

[CIT0038] Stone WS, Walser B, Gold SD, Gold PE 1991 Scopolamine-and morphine-induced impairments of spontaneous alternation performance in mice: reversal with glucose and with cholinergic and adrenergic agonists. Behav Neurol. 105:264. 10.1037//0735-7044.105.2.264 2043273

